# Phylogeographic insights into the invasion history and secondary spread of the signal crayfish in Japan

**DOI:** 10.1002/ece3.2286

**Published:** 2016-07-04

**Authors:** Nisikawa Usio, Noriko Azuma, Eric R. Larson, Cathryn L. Abbott, Julian D. Olden, Hiromi Akanuma, Kenzi Takamura, Noriko Takamura

**Affiliations:** ^1^ Institute of Nature and Environmental TechnologyKanazawa UniversityKanazawa920‐1192Japan; ^2^ Graduate School of Fisheries SciencesHokkaido UniversityHakodate 041‐8611Japan; ^3^ Department of Natural Resources and Environmental SciencesUniversity of IllinoisUrbanaIllinois61801; ^4^ School of Aquatic and Fishery SciencesUniversity of WashingtonSeattleWashington98195; ^5^ Pacific Biological StationFisheries and Oceans CanadaNanaimoBritish ColumbiaV9T 6N7Canada; ^6^ Center for Toki and Ecological RestorationNiigata UniversityNiigata950‐2181Japan; ^7^ Center for Environmental Biology and Ecosystem StudiesNational Institute for Environmental StudiesTsukuba305‐8506Japan

**Keywords:** Biological invasion, freshwater, mitochondrial DNA, *Pacifastacus leniusculus*, population genetics, propagule pressure

## Abstract

Successful invasion by nonindigenous species is often attributed to high propagule pressure, yet some foreign species become widespread despite showing reduced genetic variation due to founder effects. The signal crayfish (*Pacifastacus leniusculus*) is one such example, where rapid spread across Japan in recent decades is believed to be the result of only three founding populations. To infer the history and explore the success of this remarkable crayfish invasion, we combined detailed phylogeographical and morphological analyses conducted in both the introduced and native ranges. We sequenced 16S mitochondrial DNA of signal crayfish from across the introduced range in Japan (537 samples, 20 sites) and the native range in western North America (700 samples, 50 sites). Because chela size is often related to aggressive behavior in crayfish, and hence, their invasion success, we also measured chela size of a subset of specimens in both introduced and native ranges. Genetic diversity of introduced signal crayfish populations was as high as that of the dominant phylogeographic group in the native range, suggesting high propagule pressure during invasion. More recently established crayfish populations in Japan that originated through secondary spread from one of the founding populations exhibit reduced genetic diversity relative to older populations, probably as a result of founder effects. However, these newer populations also show larger chela size, consistent with expectations of rapid adaptations or phenotypic responses during the invasion process. Introduced signal crayfish populations in Japan originate from multiple source populations from a wide geographic range in the native range of western North America. A combination of high genetic diversity, especially for older populations in the invasive range, and rapid adaptation to colonization, manifested as larger chela in recent invasions, likely contribute to invasion success of signal crayfish in Japan.

## Introduction

Mounting evidence suggests that nonindigenous species may become successful invaders despite showing low genetic variation (Tsutsui et al. 2000; Lindholm et al. 2005). Due to the lack of a large genetic pool, genetic diversity is expected to decline following founder effects through random genetic drift or genetic bottlenecks (Lacy 1987; Dlugosch and Parker 2008a; Ficetola et al. 2008; Cristescu 2015). Nevertheless, some successful invaders exhibit evolutionary changes, phenotypic plasticity, or rapid adaptations following reduced genetic variation (Tsutsui et al. 2000; Yonekura et al. 2007; Dlugosch and Parker 2008a). In such cases, population genetics may provide useful insight into evolutionary ecology of invasive species (Leinonen et al. 2008).

Population genetics is also a powerful approach to infer the invasion history of nonindigenous species. Information on invasion history and genetic structure can help to construct management plans for problematic invaders when prevention, screening, control, or monitoring is required to mitigate their detrimental impacts on native biodiversity or ecosystem services (Sakai et al. 2001; Hampton et al. 2004). Mitochondrial DNA (mtDNA) markers have been widely used as a tool to infer the native sources, invasion pathways, genetic variation, gene flow, and demography of nonindigenous species (Ficetola et al. 2008; Gillis et al. 2009; Rollins et al. 2011). Numerous studies have reported that high propagule pressure (a large number of founders and/or multiple introductions) or genetic admixture from multiple source populations contribute to the establishment of nonindigenous species (Roman and Darling 2007; Blackburn et al. 2015).

The signal crayfish (*Pacifastacus leniusculus*) is among the world's most notorious freshwater invaders and has impacted native biodiversity throughout its introduced ranges via predation, competition, ecosystem engineering, or transmission of diseases (Nyström et al. 2001; Edgerton et al. 2004; Usio et al. 2009; Twardochleb et al. 2013). Native to the Pacific Northwest region of North America (northwest United States and southwest Canada), the signal crayfish has been introduced to 27 countries or regions in Europe and Japan for aquaculture (Usio et al. 2007; Holdich et al. 2009). A recent native to introduced range comparison of the ecology of the signal crayfish found that this species conserved its broadly omnivorous trophic function following invasion from North America, but had succeeded in establishing populations in Japan with very different climates relative to the native range (Larson et al. 2010). Furthermore, mtDNA and morphological analyses indicated that signal crayfish from their native range consisted of several cryptic lineages and some regions of the Pacific Northwest may represent recent human‐assisted invasions by this species (Larson et al. 2012). The geographic and phylogenetic origins of the invasive signal crayfish in Japan are largely unknown, but historical records indicate that a large number of signal crayfish were imported to Japan from western United States on five occasions from 1926 to 1930 (see Methods).

Earlier studies using ectosymbiont crayfish worms (Branchiobdellida (Annelida)) determined that the introduced signal crayfish in Japan consisted of three founders, that is, Lake Mashu (Hokkaido Prefecture), Tankai (Shiga Prefecture), and Akashina (Nagano Prefecture), because these populations (i.e., a group of individuals at each site) are characterized by different composition of branchiobdellidan species (Ohtaka et al. 2005; Ohtaka 2007; Nakata et al. 2010). A previous microsatellite analysis conformed to the results of the branchiobdellidan analyses (Azuma et al. 2011). Furthermore, both branchiobdellidan and microsatellite analyses indicated that Lake Mashu is the source of recent, secondary invasions of these introduced signal crayfish within Hokkaido and Honshu Islands (Nakata et al. 2010; Azuma et al. 2011). However, these previous studies only examined branchiobdellids or genetic variation of representative signal crayfish populations within the introduced range of Japan. To infer invasion history of signal crayfish in Japan, both native and introduced ranges need to be studied and contrasted.

The literature suggests that some species may succeed in the invasion process owing to rapid adaptations, evolutionary changes, or phenotypic plasticity (Dlugosch and Parker 2008b; Franks and Munshi‐South 2014). Few studies have investigated the potential for rapid adaptation among populations of invasive crayfish, but these have found that introduced populations of invasive crayfish tend to be more aggressive and grow faster than native populations of these same species (Pintor and Sih 2009; Sargent and Lodge 2014). Further, different traits or behaviors may be favoured among dispersing individuals at the periphery or leading edge of invasions relative to older core populations (Hudina et al. 2014). For example, Hudina et al. (2012) found signal crayfish at the leading edge of an invasion to display larger chela than individuals in the core population. Chela size in crayfish is highly associated with aggression, dominance, and competitive ability (Garvey and Stein 1993; Rutherford et al. 1995; Gherardi et al. 2000), and this suggests that chela size and related traits may be important to either success in, or likelihood of, dispersing and invading. Our comparison of native and invasive range signal crayfish populations provided an opportunity to also evaluate whether potential invasive traits like chelae size, and associated competitive ability and aggression, show patterns consistent with the findings above.

In this study, we use a large genetic data set from crayfish sampled in both native and introduced ranges to investigate the invasion history of the signal crayfish in Japan and make morphological comparisons between distant sites. Specifically, we tested the following hypotheses: (1) the three founding populations of the introduced signal crayfish in Japan originate from multiple sources in North America; (2) the introduced signal crayfish populations have undergone a loss of genetic diversity relative to native populations, or following successive invasions and secondary spread within Japan; and (3) recently established signal crayfish in Japan demonstrate patterns of morphological change (i.e., larger chela) consistent with expectations of increased aggression or boldness in invasive populations. Results from this study provide the first intercontinental phylogeographic comparison between the native and an introduced range for this major invasive crayfish, thereby testing whether this species has experienced reduced genetic diversity where introduced. Our results inform current management of introduction pathways and secondary spread of the signal crayfish in Japan and provide fundamental scientific insight into the genetic and morphological correlates of invasion success at biogeographical scales.

## Methods

### The native range of signal crayfish

The signal crayfish is native to the northwestern United States and southwestern Canada, including the Columbia River and its tributaries and adjacent coastal rivers. The species has also been widely introduced within the western United States, where it is a notable invasive species in the states of California and Nevada (e.g., in Lake Tahoe; Abrahamsson and Goldman 1970). Furthermore, historical records (Carl and Guiguet 1957; Bouchard 1978) and recent genetic analyses (Larson et al. 2012) suggest that portions of the assumed native range of signal crayfish may in fact represent introductions of this species for purposes including harvest or lake management. These proposed introduced regions for signal crayfish include coastal British Columbia (specifically Vancouver Island), as well as some interior Columbia River tributaries like the upper Snake River of southern Idaho. As a strong economic market for commercial harvest or aquaculture of signal crayfish grew in northern Europe and United States in the 19th century (Miller and Van Hyning 1970; Mason 1974; McGriff 1983), augmentation and translocation of this species might have also occurred within the native range. However, the introduction history of signal crayfish within North America is poorly known and merits further investigation, and it is also likely that some introduced signal crayfish in Japan originate from North American introduced sites. Accordingly, we consider all North American sites that we sampled as the native range for this comparison to Japan. Owing to the potential effects of including nonindigenous signal crayfish sites within the presumed native range in the intercontinental comparison of genetic variability (Cristescu 2015), we also repeat some of our statistical comparisons (see below) between Japan and North America using more restrictive definitions of the native range for signal crayfish.

### Introduction and range expansion of signal crayfish in Japan

From 1926 to 1930, signal crayfish were imported five times for aquaculture from western North America (Usio et al. 2007). Historical records indicate that at least 1776 individuals of signal crayfish were imported from “Portland, Oregon”, “Columbia River, Oregon”, and “Columbia, Oregon” by the former Ministry of Agricultural Forestry of Japan, 50 signal crayfish were imported (details of the origin is unknown) by a trading company (Zeikei Kyoudai Co., Kobe, Japan), and 10 signal crayfish were sold (details of the origin is unknown) by a fisheries association (Teikoku Suisankai, Japan) (Kawai et al. 2002). However, it is unclear from these records whether signal crayfish were harvested from a single or multiple locations within Oregon or elsewhere in western United States. These crayfish were subsequently introduced to three localities in Shiga Prefecture (65 individuals were introduced to Shakujinai Lake in 1926, 30 individuals were introduced to Tankai Reservoir in 1926, and 25 individuals were introduced to Taisho Pond in 1927), one locality in Hokkaido Prefecture (476 individuals were introduced to Lake Mashu in 1930), one locality in Fukui Prefecture (unknown number of individuals were introduced to Shishigaike in 1933), and one locality in Tokyo Prefecture (details of crayfish introduction are unknown). Although no official record exists, signal crayfish were possibly introduced (or escaped from the experimental station) in 1926–1930 into an irrigation stream along Sai River in Akashina town in Nagano Prefecture (Usio et al. 2007). Most of these early crayfish populations disappeared soon after introductions, but established populations from early introductions can be found in Lake Mashu (Hokkaido Prefecture), Tankai Reservoir and its inflow (Shiga Prefecture), and an irrigation stream in Akashina (Nagano Prefecture). Signal crayfish in Hokkaido have gradually expanded their range since the 1970s. At present, signal crayfish can be widely found in lentic or lotic habitats across northern and central Japan (Hokkaido, Fukushima, Fukui, Shiga, and Nagano Prefectures).

### Crayfish sampling and DNA sequencing

From 2006 to 2010, we collected signal crayfish specimens from across the introduced range in Japan and the Pacific Northwest region of North America. In the introduced range, we collected 537 signal crayfish from 20 sites across Japan. In the native range, we used 700 signal crayfish specimens from 50 sites across British Columbia, Oregon, Washington, Idaho, and northern Nevada that were published in Larson et al. (2012). We omitted from consideration in the native range two cryptic groups identified by Larson et al. (2012) that were more distinct from signal crayfish than the outgroup species used in that analysis (*Pacifastacus connectens*). Neither cryptic group, nor other species of the crayfish genus *Pacifastacus*, have been observed in Japan by our or previous studies. Therefore, in this study, new genetic data from the introduced range in Japan were used together with a portion of previously published sequence data from the native range in North America (Larson et al. 2012).

Upon collection, a cheliped or a walking leg was clipped from each crayfish and preserved in 100% ethanol. For juvenile crayfish, whole specimens were either preserved in 100% ethanol or immediately frozen following live transport from the sample site. Total genomic DNA was extracted from tissue samples dissected from the abdomen, chelipeds, or walking legs using the DNeasy Tissue Kit (Qiagen Inc., Valencia, CA). Using the 16Sar‐L and 16Sbr‐H primers (Imai et al. 2004), we amplified and sequenced a partial region (437–440 bp) of the 16S ribosomal RNA gene in mtDNA as described in Larson et al.(2012). Editing and assembly of contigs were completed using ContigExpress version 11 (Invitrogen Corporation, Carlsbad, CA). Sequences were aligned in BioEdit version 7.1.3.0 (Hall 1999).

All sequences found in the native range have been previously deposited in GenBank (Larson et al. 2012; see Table S1 for correspondence between each haplotype and the accession number). In this study, we deposited in GenBank the sequence of one additional haplotype that was only found in Japan (HapK, accession no. LC081181).

### Morphological analysis

We made morphological measurements on 323 crayfish from 17 introduced sites in Japan (mean 19 individuals/site, range 11–20) and 128 crayfish from 23 sites in the Pacific Northwest native range (mean 7 individuals/site, range 3–22). As for the genetic data, morphological data from the introduced range in Japan are newly reported in this study, while those from the native range in North America use previously published data from Larson et al. (2012).

Morphological measurements were made using Vernier callipers to 0.01 mm. Only male crayfish with ≥20 mm carapace length were used in our morphological analysis because crayfish chela tends to be larger in males relative to females (Stein 1976) and this size cutoff is consistent with past definitions of adult crayfish (Larson et al. 2012). We obtained chela area of each crayfish by approximating the right chela to a triangle (chela area = chela length × chela width × 1/2). When the right chela was missing or showed signs of regeneration, we measured the left chela. We standardized chela area as a ratio to carapace length (ChA.CL) to account for size differences among individual crayfish. Unfortunately, owing to the storage procedure of the crayfish specimens, most specimens were not labeled individually and, consequently, morphological results could not be paired with genetic results for each crayfish. We therefore evaluated the relationship between genetic diversity and ChA.CL using the mean value of ChA.CL at each site, when we tested for potential effect of genetic admixture on chela size in the introduced signal crayfish populations in Japan.

### Data analysis

We used the program TCS 1.21 (Clement et al. 2000) to construct a 95% statistical parsimony cladogram network to visualize the phylogenetic relationships among haplotypes. Loops in the network were manually resolved following rules established in accordance with the coalescent theory (Pfenninger and Posada 2002).

On the basis of the Akaike information criterion (AIC), we performed jModeltest 2.1.8 to select the best model for DNA sequence evolution of among‐site variation (Guindon and Gascuel 2003; Darriba et al. 2012). Consequently, we selected the Kimura 2‐parameter evolution model with gamma correction (K2P + G) (*γ* = 0.03) for use in subsequent spatial analysis of molecular variance (SAMOVA). To identify best genetic groups that are maximally differentiated from each other, we performed SAMOVA in SAMOVA 2.0 for all sites in the introduced and native ranges (Dupanloup et al. 2002). We compared the *φ*_CT_ statistic for the number of groups (*K*) ranging from 2 to 10 without geographic constraints with 100 annealing processes as starting conditions. We determined the most likely number of groups when *φ*_CT_ reached a plateau (Dupanloup et al. 2002). Using the Kimura 2‐parameter evolution model with gamma correction (*γ* = 0.03), we subsequently performed analysis of molecular variance (AMOVA) in Arlequin ver. 3.5.2.2 (Excoffier and Lischer 2010) to measure the amount of genetic covariation between the groups suggested by SAMOVA. We evaluated the significance of the *F*‐statistics by running 10,000 permutations of the data set. When the groups comprised only one site, we calculated *φ*_ST_ values in Arlequin as a measure of pairwise genetic differences between the introduced and native groups. We did not estimate the significance of covariation between groups for those consisting of only one site because of inadequate replication.

To infer changes in genetic diversity following crayfish introductions, we calculated the number of haplotypes (*N*_h_), haplotype diversity (*h*), and nucleotide diversity (*π*) in each site in introduced and native ranges. We calculated the genetic diversity indices in Arlequin for the sites comprising three or more individual crayfish samples or specimens. To test for differences in *N*_h_, *h,* and *π* between signal crayfish populations in introduced and native ranges, we conducted Wilcoxon rank‐sum tests using R version 3.2.0 (R Development Core Team, 2015). Owing to the potential effect of nonindigenous populations in the Pacific Northwest on our native to introduced range comparisons, we repeated these analyses with more restrictive definitions of the Pacific Northwest native range (per Larson et al. 2012) to test the sensitivity of our results to native range definition (see Table 1).

**Table 1:**
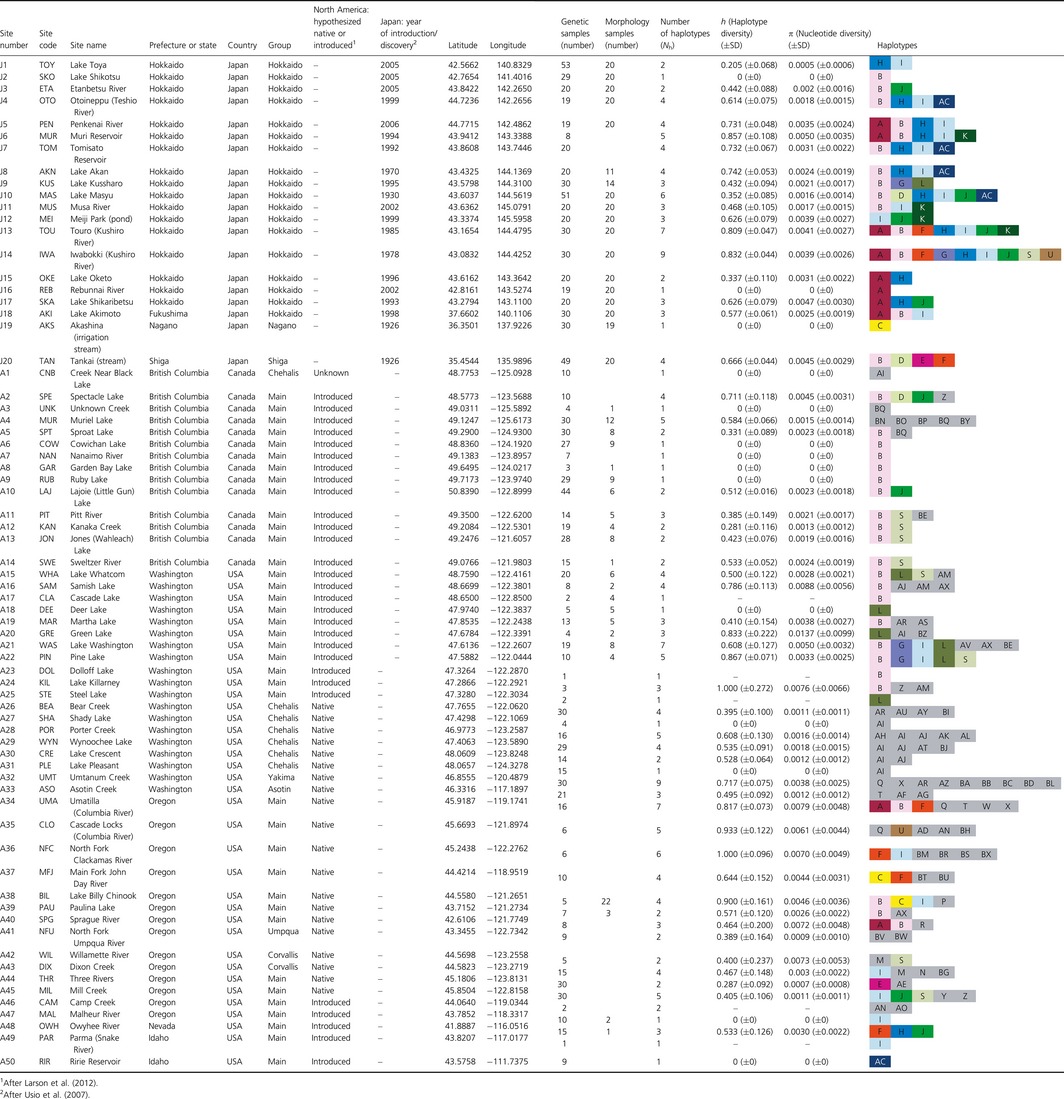
Sites of the signal crayfish (*Pacifastacus leniusculus*) sampled in introduced (Japan) and native ranges (southwest Canada and northwest United States) with genetic and morphological sample numbers, descriptive statistics of genetic diversity, and the haplotypes found at each site. Haplotypes found in Japan are in color, whereas those found only in North America are in gray

We investigated whether ChA.CL differs between introduced populations and their putative source populations in the native range. As in the genetic diversity calculations, we only included the sites comprising three or more samples in the morphological analyses. We used a linear mixed‐effects model in the R package lmerTest (Kuznetsova et al. 2016), with range (introduced or native) as a fixed factor and site identity nested within the range as a random factor, to compare the mean difference in ChA.CL between the introduced and native range signal crayfish populations. We did not evaluate the effect of native range definition on the ChA.CL comparison owing to prohibitively low sample sizes for morphological measurements in some areas of the Pacific Northwest range. We subsequently used a linear mixed‐effects model in R, with site identity as a random factor and year of introduction/discovery as a fixed factor, to investigate the relationships between year of introduction/discovery and ChA.CL in the introduced range. We also performed ordinary least‐square regression analysis in R to investigate the relationship between number of haplotypes and ChA.CL in the introduced populations. We performed the linear mixed‐effects model and regression analyses both including and excluding the introduced Nagano and Shiga populations, as these two populations have been shown to be confined to their original introduction sites and haplotype composition in these populations differed relative to Lake Mashu and the secondary introduction sites from this lake (see Results). When the normality assumption of the model residual could not be met, we applied log transformation to the independent variable.

## Results

**Figure 1:**
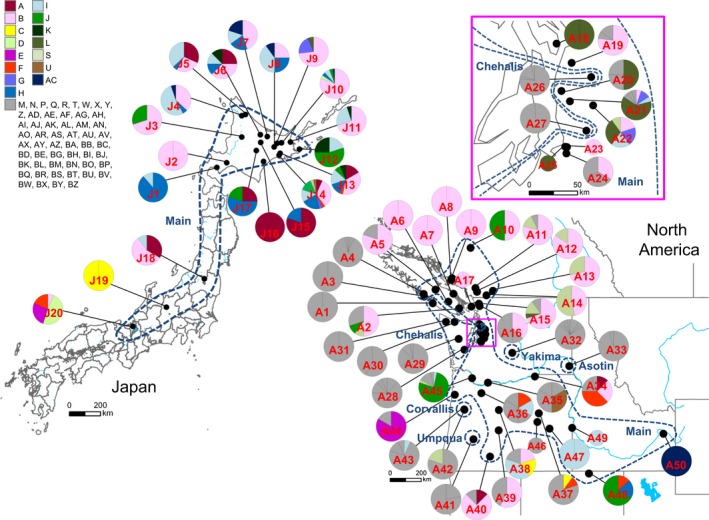
Geographic distribution and haplotypes observed in signal crayfish (*Pacifastacus leniusculus*) populations in Japan and North America. Smaller circles (A17, A23, A25, A46, and A49) indicate smaller sample sizes (<3 individuals). The genetic groups of the introduced and native range populations are delineated by blue broken lines. Haplotypes found in Japan are in color, whereas those found only in North America are in gray. See Table 1 for site numbers.

**Figure 2:**
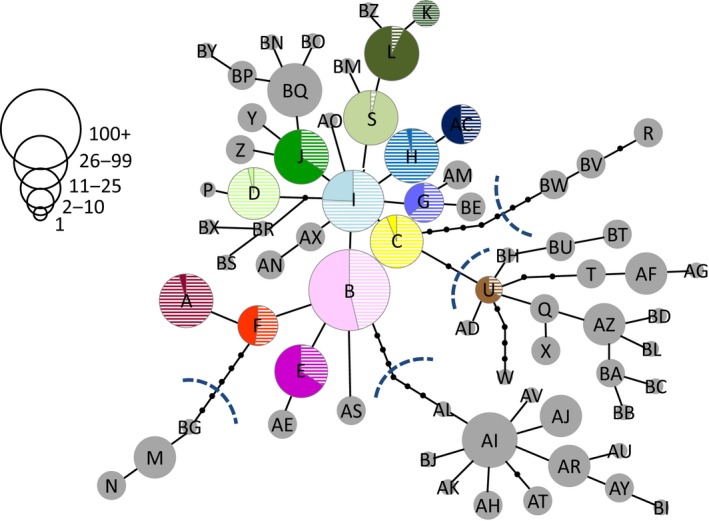
Statistical parsimony haplotype network of signal crayfish (*Pacifastacus leniusculus*) samples from Japan and North America. The genetic lineages are delineated by blue broken lines. Haplotypes found both in Japan (indicated by horizontal stripes) and North America (filled) are in color, whereas those found only in North America are in gray. Black dots indicate missing or unsampled haplotypes. The size of the circles is proportional to frequency. Each link between haplotypes represents one mutation step.

Our mtDNA analysis revealed 15 different haplotypes in the 20 introduced sites of signal crayfish in Japan and 69 haplotypes in the 50 native range sites (Table 1, Fig. 1). Fourteen haplotypes identified in Japan were found in 37 of 50 sites (74%) in the native range; nine haplotypes occurred in the more restrictive native range hypothesized by Larson et al. (2012), while five haplotypes occurred in the hypothesized introduced range in North America. All haplotypes from Japan except K were found in what we identified as the Main native range group, and two haplotypes were also found at the Corvallis sites (Figs. 1, 2). However, haplotype K, found in four introduced sites in Japan (J6, J11, J12, and J13), was not identified in the native range sites. In 17 of 20 introduced sites in Japan (85%), two or more haplotypes were detected; only three introduced populations (J2, J16, and J19) were monomorphic.

The haplotype composition differed among the three founding populations in Japan. Signal crayfish in Lake Mashu had seven haplotypes, those in Akashina had only one haplotype, and those in Tankai had four haplotypes. Although haplotypes B and D were found in both Lake Mashu and Tankai, the remaining 2–4 haplotypes differed between the two populations. Haplotype C was only found in Akashina. Except for Akashina, where the population comprised only one haplotype, haplotypes from different or multiple native range sites were likely introduced to Lake Mashu and Tankai. For example, haplotypes H, I, and AC, identified in Lake Mashu, were not found sympatrically in the native range. Likewise, haplotypes D, E, and F, identified in Tankai, did not co‐occur in any of the native range sites.

Among the three founding populations in Japan, the number of haplotypes was greatest in Lake Mashu, whereas haplotype diversity and nucleotide diversity were greatest in Tankai (Table 1). Only one haplotype was found from Akashina, and consequently, haplotype and nucleotide diversity were zero at this site. Although historical records show that Kushiro River populations originated from Lake Mashu (Usio et al. 2007), these secondary invasions (J13 and J14) had a greater number of haplotypes (*N*_h_ = 7–9), haplotype diversity (*h *=* *0.81–0.83), and nucleotide diversity (*π* = 0.0039–0.0041) relative to the initial or founding population (*N*_h_ = 6, *h *=* *0.35, *π *= 0.0016). Nine recently invaded sites (J5, J6, J9, J11, J12, J15, J16, J17, and J18) in Hokkaido and Fukushima Prefectures contained haplotypes A, G, and/or K, which were also found in Touro or Iwabokki but not in Lake Mashu. Therefore, the Kushiro River probably acted as a source for tertiary invasions to these sites.

The haplotype network showed that the native signal crayfish populations consisted of five lineages which were connected by one to seven missing haplotypes (i.e., nonsampled or extinct haplotypes; Fig. 2). The largest lineage, which we defined as the Main group (above), consisted of 34 haplotypes including the most prevalent haplotype B, and the haplotypes in this lineage were found across the native range. A second lineage consisted of three haplotypes (BG, M, and N) that were collected from the Corvallis region in west central Oregon. A third lineage, identified as the Chehalis group in Larson et al. (2012), consisted of 12 haplotypes (including AI), and the haplotypes in this lineage were in western Washington and from one location on Vancouver Island, British Columbia. A fourth lineage includes seventeen haplotypes (including U) that were found from the Columbia River and its tributaries east of the Cascade Mountains. Finally, a fifth lineage consisted of three haplotypes (BW, BV, and R) that were found from Umpqua and Klamath River tributaries in southwest Oregon. Except for the haplotype U, all haplotypes identified in Japan are from the most common lineage.

In SAMOVA, *φ*_CT_ increased to a plateau or asymptote at six clusters (*K* = 6; Table 2, Fig. S1), identifying six genetic groups. When single‐site groups were not considered as independent genetic groups, two clusters (*K* = 2) were selected. In both cases, all introduced populations were clustered into the same group as the Main group in the Pacific Northwest. Subsequent pairwise AMOVAs or pairwise population differentiation tests (when only one population consisted of each group) showed high *φ*_CT_ or *φ*_ST_ between the six genetic groups (Table 3). When AMOVA was performed between Japanese and North American sites within the Main genetic group, percentage of covariance did not differ between the subgroups (*φ*_CT_ = 0.014, *P *=* *0.182 ± 0.003). Thus, all introduced populations in Japan likely originate from the Main group in the native range.

**Table 2:** Summary results of spatial analysis of molecular variance (SAMOVA) using the Kimura 2‐parameter evolution model with gamma correction (*γ* = 0.03). See Table 1 for site numbers

*K*	Group composition										*φ*_CT_
2	[J1, J2, J3, J4, J5, J6, J7, J8, J9, J10, J11, J12, J13, J14, J15, J16, J17, J18, J19, J20, A2, A3, A4, A5, A6, A7, A8, A9, A10, A11, A12, A13, A14, A15, A16, A18, A19, A20, A21, A22, A24, A32, A33, A34, A35, A36, A37, A38, A39, A40, A41, A42, A43, A44, A45, A47, A48, A50]	[A1, A26, A27, A28, A29, A30, A31]									0.838
3	[J1, J2, J3, J4, J5, J6, J7, J8, J9, J10, J11, J12, J13, J14, J15, J16, J17, J18, J19, J20, A2, A3, A4, A5, A6, A7, A8, A9, A10, A11, A12, A13, A14, A15, A16, A18, A19, A20, A21, A22, A24, A32, A33, A34, A35, A36, A37, A38, A39, A40, A42, A43, A44, A45, A47, A48, A50]	[A1, A26, A27, A28, A29, A30, A31]	[A41]								0.851
4	[J1, J2, J3, J4, J5, J6, J7, J8, J9, J10, J11, J12, J13, J14, J15, J16, J17, J18, J19, J20, A2, A3, A4, A5, A6, A7, A8, A9, A10, A11, A12, A13, A14, A15, A16, A18, A19, A20, A21, A22, A24, A32, A34, A35, A36, A37, A38, A39, A40, A42, A43, A44, A45, A47, A48, A50]	[A1, A26, A27, A28, A29, A30, A31]	[A41]	[A33]							0.861
5	[J1, J2, J3, J4, J5, J6, J7, J8, J9, J10, J11, J12, J13, J14, J15, J16, J17, J18, J19, J20, A2, A3, A4, A5, A6, A7, A8, A9, A10, A11, A12, A13, A14, A15, A16, A18, A19, A20, A21, A22, A24, A34, A35, A36, A37, A38, A39, A40, A42, A43, A44, A45, A47, A48, A50]	[A1, A26, A27, A28, A29, A30, A31]	[A41]	[A33]	[A42, A43]						0.868
6	[J1, J2, J3, J4, J5, J6, J7, J8, J9, 10, JJ11, J12, J13, J14, J15, J16, J17, J18, J19, J20, A2, A3, A4, A5, A6, A7, A8, A9, A10, A11, A12, A13, A14, A15, A16, A18, A19, A20, A21, A22, A24, A34, A35, A36, A37, A38, A39, A40, A44, A45, A47, A48, A50]	[A1, A26, A27, A28, A29, A30, A31]	[A41]	[A33]	[A42, A43]	[A32]					0.877
7	[J1, J2, J3, J4, J5, J6, J7, J8, J9, J10, J11, J12, J13, J14, J15, J16, J17, J18, J19, J20, A2, A3, A4, A5, A6, A7, A8, A9, A10, A11, A12, A13, A14, A15, A16, A18, A19, A20, A21, A22, A24, A34, A35, A36, A37, A38, A39, A40, A44, A45, A47, A48, A50]	[A1, A27, A28, A29, A30, A31]	[A41]	[A33]	[A42, A43]	[A32]	[A26]				0.877
8	[J1, J2, J3, J4, J5, J6, J7, J8, J9, J10, J11, J12, J13, J14, J15, J16, J17, J18, J19, J20, A2, A3, A4, A5, A6, A7, A8, A9, A10, A11, A12, A13, A14, A15, A16, A18, A19, A20, A21, A22, A24, A34, A35, A36, A38, A39, A40, A44, A45, A47, A48, A50]	[A1, A27, A28, A29, A30, A31]	[A41]	[A33]	[A42, A43]	[A32]	[A26]	[A37]			0.878
9	[J1, J2, J3, J4, J5, J6, J7, J8, J9, J10, J11, J12, J13, J14, J15, J16, J17, J18, J19, J20, A2, A3, A4, A5, A6, A7, A8, A9, A10, A11, A12, A13, A14, A15, A16, A18, A19, A20, A21, A22, A24, A34, A36, A38, A39, A40, A44, A45, A47, A48, A50]	[A1, A27, A28, A29, A30, A31]	[A41]	[A33]	[A42, A43]	[A32]	[A26]	[A37]	[A35]		0.877
10	[J1, J2, J3, J4, J5, J6, J7, J8, J9, J10, J11, J12, J13, J14, J15, J16, J17, J18, J19, J20, A2, A4, A5, A6, A7, A8, A9, A10, A11, A12, A13, A14, A15, A16, A18, A19, A20, A21, A22, A24, A34, A36, A38, A39, A40, A44, A45, A47, A48, A50]	[A1, A27, A28, A29, A30, A31]	[A41]	[A33]	[A42, A43]	[A32]	[A26]	[A37]	[A35]	[A3]	0.877

**Table 3:** Results of AMOVAs evaluating the amount of genetic covariance (based on *φ*_CT_ or *φ*_ST_) between groups of introduced and native range signal crayfish

		Main	Chehalis	Umpqua	Asotin	Yakima	Corvallis
		J1, J2, J3, J4, J5, J6, J7, J8, J9, J10, J11, J12, J13, J14, J15, J16, J17, J18, J19, J20, A2, A3, A4, A5, A6, A7, A8, A9, A10, A11, A12, A13, A14, A15, A16, A18, A19, A20, A21, A22, A24, A34, A36, A38, A39, A40, A44, A45, A47, A48, A50	A1, A26, A27, A28, A29, A30, A31	A41	A33	A32	A42, A43
Main	J1, J2, J3, J4, J5, J6, J7, J8, J9, J10, J11, J12, J13, J14, J15, J16, J17, J18, J19, J20, A2, A3, A4, A5, A6, A7, A8, A9, A10, A11, A12, A13, A14, A15, A16, A18, A19, A20, A21, A22, A24, A34, A36, A38, A39, A40, A44, A45, A47, A48, A50	**–**					
Chehalis	A1, A26, A27, A28, A29, A30, A31	0.897***	**–**				
Umpqua	A41	0.900n.d.	0.972n.d.	**–**			
Asotin	A33	0.835n.d.	0.909n.d.	0.989***	**–**		
Yakima	A32	0.748n.d.	0.942n.d.	0.939***	0.843***	**–**	
Corvallis	A42, A43	0.790***	0.938*	0.899n.d.	0.921n.d.	0.880n.d.	**–**

Genetic covariance was expressed in percentages. Six genetic groups were identified in the native range on the basis of spatial analysis of molecular variance (SAMOVA). The significance of covariation among groups was not estimated for the groups comprising only one population because of low statistical power. See Table 1 for site numbers.

*P <* *0.05, ***P <* *0.001, n.d., not determined.

The number of haplotypes (*N*_h_) in introduced signal crayfish populations in Japan (interquartile range: 2.00–4.00, median = 3.00) was as high as that of all sites in the native range (interquartile range: 2.00–4.00, median = 3.00; Wilcoxon rank‐sum test, *W *=* *514.5, *P *=* *0.35) (Fig. S2A) or the Main group in the native range (interquartile range: 2.00–4.00, median = 3.00) (*W *=* *377.5, *P *=* *0.38) (Fig. S2B). Haplotype diversity (*h*) was not significantly different between introduced signal crayfish populations in Japan (interquartile range: 0.348–0.731, median = 0.596) and all native range populations (interquartile range: 0.281–0.608, median=0.467; Wilcoxon rank‐sum test, *W *=* *516.5, *P *=* *0.35) or native range populations belonging to the Main group (interquartile range: 0.281–0.711, median = 0.500; Wilcoxon rank‐sum test, *W *=* *358.0, *P *=* *0.61). Likewise, there was no statistical difference in nucleotide diversity (*π*) between the introduced Japanese populations (interquartile range: 0.0017–0.0039, median = 0.0025) and all groups in the native range (interquartile range: 0.0007–0.0044, median = 0.0021; Wilcoxon rank‐sum test, *W *=* *480.5, *P *=* *0.67) or the Main group populations in the native range (interquartile range: 0.0007–0.0046, median = 0.0024; Wilcoxon rank‐sum test, *W *=* *317.0, *P *=* *0.82). These results were not generally sensitive to inclusion of all Pacific Northwest signal crayfish genetic groups or the use of a more restrictive native range definition (Fig. S2C, D). The only exception was that *π* of signal crayfish was higher in the Main group in the restrictive native range relative to that in Japan (Fig. S2D).

**Figure 3:**
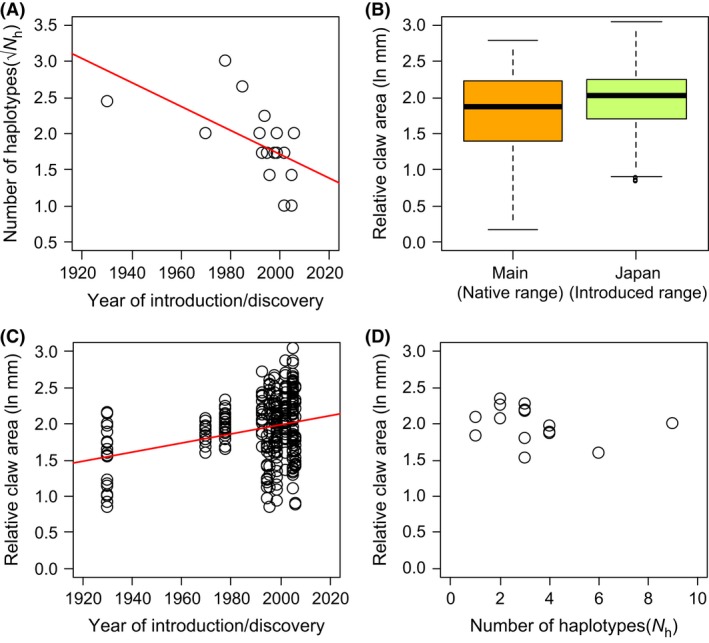
(A) The relationship between the number of haplotypes (*N*_h_) and the year of introduction or discovery for each signal crayfish population of the Hokkaido group (*y* = −0.0167√*x* + 35.057, *r *= −0.581, *P *=* *0.011). (B) Mean (±1SE) chela area to carapace length ratio (ChA.CL) of signal crayfish in the native range Main group and Japan (introduced range) (linear mixed‐effects model: *P *=* *0.049). (C) The relationship between ChA.CL and the year of introduction or discovery in the Hokkaido group (linear mixed‐effects model: *t *=* *2.141, *P *=* *0.049). (D) The relationship between ChA.CL and the number of haplotypes in the Hokkaido group (*r *= −0.293, *P *=* *0.28).

When the number of haplotypes in the introduced populations in Japan was regressed against the year of establishment or discovery, there was no significant relationship between these two variables (*r *= −0.197, *P *=* *0.40). However, there was a significant negative relationship between the number of haplotypes and the year of establishment or discovery when only specimens of the Hokkaido (or Lake Mashu originating) group were considered in the analysis (*r *= −0.581, *P *=* *0.011; Fig. 3A).

Morphological analysis showed that the average ChA.CL was greater in the introduced Japanese populations relative to that of the Main group in the native range (linear mixed‐effects model: *t *= −2.047, *P *=* *0.049; Fig. 3B). We did not find a significant relationship between ChA.CL and the year of establishment or discovery when all specimens of the introduced groups were included in the analysis (linear mixed‐effects model: *t *=* *0.895, *P *=* *0.38). However, we found a significant positive relationship between ChA.CL and the year of establishment or discovery when only specimens of the introduced Hokkaido group were considered in the analysis (linear mixed‐effects model: *t *=* *2.141, *P *=* *0.049; Fig. 3C). There was no significant relationship between ChA.CL and the number of haplotypes in all introduced groups (*r *= −0.197, *P *=* *0.40) or that in the Hokkaido group (*r *= −0.293, *P *=* *0.29; Fig. 3D).

## Discussion

We found that the introduced signal crayfish populations in Japan originate from multiple source populations from the most widely distributed genetic group in the native range, encompassing British Columbia, Washington, Oregon, Idaho, and northern Nevada. The differences in haplotype composition among the three founding populations in Japan are likely the consequences of founder effects (Akashina) or genetic admixture (Lake Mashu and Tankai), as suggested from the distributions of haplotypes in the native range and SAMOVA grouping. Some of these putative source populations from the Pacific Northwest to Japan may themselves be introductions (Larson et al. 2012). Furthermore, we cannot rule out the possibility that the six haplotypes that were only found in Japan and/or the hypothesized introduced range in North America might also occur along the lower Columbia River (or elsewhere) because of our relatively low sampling effort in that specific region, which is a likely source for some of the earliest signal crayfish translocations dating back to the late 19th century (Miller and Van Hyning 1970).

In theory, invasive populations are expected to suffer from loss of genetic variation due to founder effects, genetic bottlenecks, and genetic drift. In contrast to these expectations, studies investigating genetic diversity of aquatic species often show little sign of reduced genetic variation following biological invasions, perhaps because biological invasions in aquatic ecosystems are often associated with high propagule pressure (reviewed in Roman and Darling 2007). In addition, multiple introductions from disparate native range sources may result in genetic admixture, which might enhance the chance for nonindigenous species to establish in a new environment in some cases (but see Cristescu 2015 for other outcomes of admixture and genetic diversity on invasion success). In our study, genetic admixture from multiple source populations within the native range of the Main group may have contributed to high genetic diversity in the introduced signal crayfish populations in Japan. Consequently, genetic diversity indices (*N*_h_, *h* and *π*) of the introduced signal crayfish were as high as those of the Main group in the native range regardless of whether or not we consider a more restrictive or expansive native range classification.

We also found that signal crayfish in Japan have larger chela (ChA.CL) relative to their putative source populations in the native range, even if some of these native range populations (e.g., British Columbia) may also represent human introductions of the species within the Pacific Northwest. Furthermore, there was a positive relationship between chela size and the year of establishment or discovery in the Hokkaido introduced group. An increase in the size of crayfish chelae in recently introduced populations may be a response to interactions with conspecific predators/competitors, avian, mammal, or fish predators or other biotic or abiotic factors. In particular, crayfish with large chela are expected to have advantages in acquiring limited resources, such as food, shelter, and mates, because dominance hierarchy or survivorship in crayfish is largely determined by chela size (Garvey and Stein 1993; Rutherford et al. 1995; Gherardi et al. 2000). Together, these findings support past studies that have found invasive populations of signal crayfish to be more aggressive than native populations (Pintor et al. 2008) and observed dispersing or peripheral signal crayfish to have larger chela than crayfish in the older, core of an invasive population (Hudina et al. 2012). Species invasions provide opportunities for rapid adaptation to new environments, and spreading invaders can have spatially structured distributions of adaptive functional traits or behaviors (Phillips et al. 2010; Hudina et al. 2014). Our findings suggest that impacts of signal crayfish on native species and ecosystems in Japan may be related to rapid adaptation or behavioral change associated with invasion (Pintor et al. 2009; Sargent and Lodge 2014), and we believe this area merits more investigation to both mitigate the effects of invasive signal crayfish and better understand the success of some invaders.

Historical records and a previous microsatellite analysis indicated that the Hokkaido signal crayfish group originated from the Lake Mashu population of this island (Usio et al. 2007; Azuma et al. 2011). However, only anecdotal evidence supports crayfish transport out of Lake Mashu. Lake Mashu is an ultra‐oligotrophic caldera lake without inflow or outflow streams and is surrounded by 150‐ to 350‐m cliffs and steep slopes. It is extremely unlikely that signal crayfish dispersed over land, although human access to the lake is also restricted because Lake Mashu is a special protected area of Akan National Park. Regardless, we believe that secondary spread of signal crayfish out of Lake Mashu to regions such as the Kushiro River was probably made by intentional, illegal translocations by anglers or through accidental translocation with other stocked fish originating from Lake Mashu. Furthermore, the lead author has observed unintentional translocation of signal crayfish with macrophyte restoration activities in the Kushiro River basin. At present, the keeping, rearing, transporting, translocating, and selling of live signal crayfish are restricted under the Invasive Alien Species Act, but much attention should be paid on unintentional introductions with fish stocks or macrophytes from invaded water bodies.

High genetic diversity at the locations of early signal crayfish introductions in Hokkaido (Lake Mashu, Iwabokki, and Touro) may have contributed to the subsequent success of this species and its widespread distribution in Japan. However, younger populations produced by secondary spread or subsequent introductions within Japan generally have lower genetic diversity, and high genetic variation or admixture from multiple source populations does not seem to be a prerequisite for invasion success in this species (Cristescu 2015). Invasive populations of another highly invasive crayfish, red swamp crayfish *Procambarus clarkii*, often have low genetic diversity in their introduced ranges and show reductions in genetic diversity with secondary spread (Torres and Alvarez 2012; Paulson and Martin 2014). Some studies have found evidence of rapid adaptations following loss of genetic variation (Tsutsui et al. 2000; Yonekura et al. 2007; Dlugosch and Parker 2008a). In our study, reduced genetic variability in recently established signal crayfish populations was evident from the Hokkaido group, with the exceptions of two Kushiro River populations (Touro and Iwabokki) that probably originate from Lake Mashu, and some of the most recently established populations (Rebunnai River and Lake Shikotsu) are monomorphic. Acquisition of adaptive traits such as large chela may explain success of signal crayfish in secondary invasions, although studies on the behavior and ecological interactions of peripheral and core populations are needed.

To date, we have established that invasive signal crayfish in Japan have multiple native range source populations and that high genetic diversity associated with this admixture in older invasive populations in the Hokkaido group attenuates to lower genetic diversity in younger populations associated with secondary spread or subsequent introductions within the country. Chela size, which is associated with aggressive behavior and competitive dominance in crayfish, tends to be larger in the invasive range than native range for signal crayfish and has a tendency to become larger in newer relative to older populations within Japan. Related to the potential for rapid adaptation within invasive range populations of signal crayfish, we previously showed that although the broadly omnivorous trophic function of this species is conserved between its native and invasive range, this crayfish has established in very distinct climates in Japan relative to the Pacific Northwest (Larson et al. 2010). More resolved studies are needed to address mechanisms of potential rapid adaptation within this species toward broad understanding of success of signal crayfish invasions and development of management strategies for such invaders.

## Conflict of Interest

None declared.

## Supporting information


**Figure S1.** The relationship between fixation index (*φ*_CT_) and number of clusters (*K*) in the signal crayfish in Japan and North America based on spatial analysis of molecular variance (SAMOVA) using the Kimura 2‐parameter evolution model with gamma correction (gamma = 0.03).
**Figure S2.** Comparisons of genetic diversity indices (*N*_h_, *h* and *π*) of signal crayfish between North America and Japan when restrictive native range definitions and genetic groups are considered (C and D) or not (A and B).

**Table S1.** Correspondence between each 16S mitochondrial DNA haplotype and the GenBank accession number of signal crayfish (*Pacifastacus leniusculus*) in North America and Japan.
