# Supplementary material for: Phylogeographic insights into the invasion history and secondary spread of the signal crayfish in Japan
Source: Ecol Evol. 2016 Jul 4;6(15):5366–82. doi: 10.1002/ece3.2286 (PMC4984510; doi:10.1002/ece3.2286)
Supplement: Supplementary file 1 — Figure S1. The relationship between fixation index (φ CT) and number of clusters (K) in the signal crayfish in Japan and North America based on spatial analysis of molecular variance (SAMOVA) using the Kimura 2‐parameter evolution model with gamma correction (gamma = 0.03). Figure S2. Comparisons of genetic diversity indices (N h, h and π) of signal crayfish between North America and Japan when restrictive native range definitions and genetic groups are considered (C and D) or not (A and B). [file ECE3-6-5366-s001.pptx]

## Slide 1
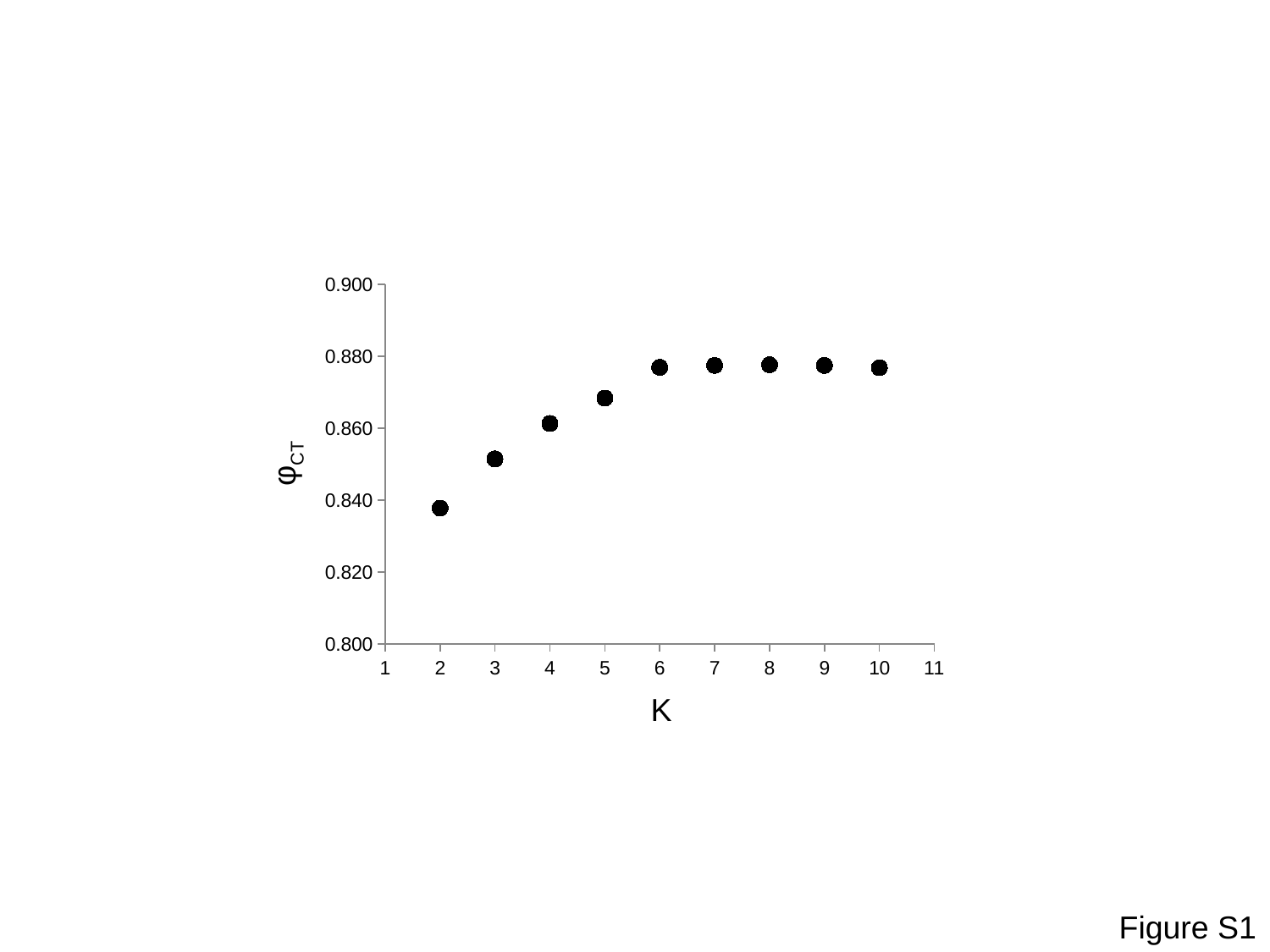

### Chart
| Category | |
|---|---|φCT
K
Figure S1

## Slide 2
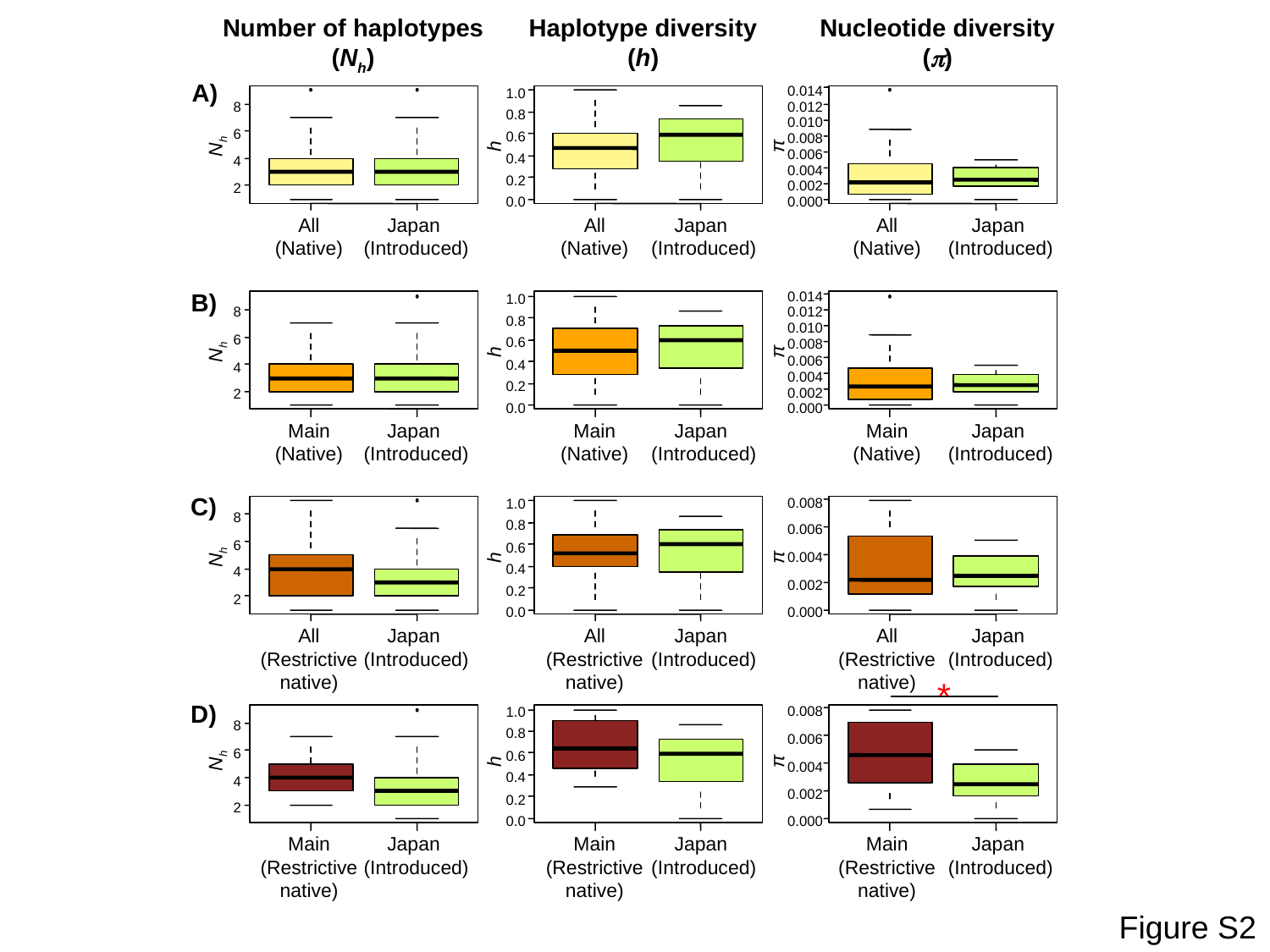

Number of haplotypes
(Nh)
Haplotype diversity
(h)
Nucleotide diversity
(π)
A)
0.014
1.0
8
0.012
0.8
0.010
6
0.6
0.008
π
Nh
h
0.006
0.4
4
0.004
0.2
0.002
2
0.0
0.000
All
(Native)
Japan
(Introduced)
All
(Native)
Japan
(Introduced)
All
(Native)
Japan
(Introduced)
B)
0.014
1.0
8
0.012
0.8
0.010
6
0.6
0.008
π
Nh
h
0.006
0.4
4
0.004
0.2
0.002
2
0.0
0.000
Main
(Native)
Japan
(Introduced)
Main
(Native)
Japan
(Introduced)
Main
(Native)
Japan
(Introduced)
C)
0.008
1.0
8
0.8
0.006
6
0.6
0.004
Nh
h
π
0.4
4
0.002
0.2
2
0.0
0.000
All
(Restrictive
native)
Japan
(Introduced)
All
(Restrictive
native)
Japan
(Introduced)
All
(Restrictive
native)
Japan
(Introduced)
*
D)
0.008
1.0
8
0.8
0.006
6
0.6
Nh
h
π
0.004
0.4
4
0.002
0.2
2
0.0
0.000
Main
(Restrictive
native)
Japan
(Introduced)
Main
(Restrictive
native)
Japan
(Introduced)
Main
(Restrictive
native)
Japan
(Introduced)
Figure S2
